# Factors Influencing Patient Preference for Surgical Intervention Versus Medical Therapy Among Cambodian Adults With Advanced Glaucoma: A Health Belief Model-Based Analysis

**DOI:** 10.7759/cureus.102403

**Published:** 2026-01-27

**Authors:** Channdarith Kith, Amarin Mar, Piseth Kong, Bunseng Sea, Kossama Chukmol, Ratneary Hav, Saly Saint

**Affiliations:** 1 Faculty of Medicine, University of Health Sciences, Phnom Penh, KHM; 2 Department of Ophthalmology, Preah Ang Duong Hospital, Phnom Penh, KHM; 3 College of Medicine and Veterinary Medicine, The University of Edinburgh, Edinburgh, GBR

**Keywords:** advanced glaucoma, health beliefs, medical therapy, surgical intervention, treatment preferences

## Abstract

Objectives

Glaucoma is the major cause of irreversible blindness globally and ranks as the sixth leading cause of avoidable blindness in Cambodia. Recent advancements in glaucoma treatment techniques have emerged; yet, patient treatment preferences, particularly within low-resource environments, remain poorly understood. We are using the Health Belief Model (HBM) to identify factors that influence the preference for surgical versus medical therapy in adults with advanced glaucoma in Cambodia.

Methods

A hospital-based cross-sectional study was conducted at Preah Ang Duong Hospital, Phnom Penh, Cambodia, between January and June 2024. A structured, validated questionnaire evaluated sociodemographic characteristics, clinical data, glaucoma awareness, and HBM constructs (susceptibility, severity, benefits, barriers, self-efficacy, and cues to action). Logistic regression was used to determine the independent factors of surgical preference.

Results

Among the 487 participants, merely 138 of them (28.13%) favored surgical intervention. Patients who chose surgery had fewer perceived barriers, stronger perceptions of severity, susceptibility, benefits, and self-efficacy, and considerably higher knowledge scores (p=0.048). Multivariable analysis indicated that perceived benefits (AOR=1.51; 95% CI: 1.16-1.94), perceived severity (AOR=1.71; 95% CI: 1.17-2.51), low perceived barriers (AOR=0.61; 95% CI: 0.49-0.77), and high self-efficacy (AOR=1.46; 95% CI: 1.11-1.93) were independent predictors of surgical preference.

Conclusion

Psychological and contextual factors significantly influence treatment decisions among Cambodian patients with advanced glaucoma in our clinical setting. Enhancing knowledge, addressing misconceptions about surgery, and improving access to glaucoma services may increase acceptance of surgical intervention, promote better disease management, and subsequently help prevent blindness in our community.

## Introduction

Glaucoma is the leading cause of irreversible blindness globally, and it is becoming a major public health concern, particularly among the elderly population. In 2020, glaucoma accounted for over 11% of worldwide blindness in those aged 50 and above, with an estimated 3.6 million individuals becoming blind from the condition [1,2]. In Cambodia, national data from the Rapid Assessment of Avoidable Blindness (RAAB) reveal that glaucoma is one of the primary causes of irreversible blindness [3], but its actual prevalence is probably underestimated due to underdiagnosis [4,5].

Reducing intraocular pressure is the main objective of glaucoma treatment to stop further optic nerve damage and loss of visual field [6,7]. Although medical therapy is usually used as the first-line treatment, surgical intervention is often recommended for advanced cases to attain long-term pressure control and decrease reliance on medication [8,9]. In our clinical setting, even at advanced stages, many patients still prefer lifetime medical therapy despite evidence that surgical management is effective.

Patient treatment preference is widely acknowledged as a vital factor influencing adherence, satisfaction, and long-term outcomes. The Health Belief Model (HBM) offers a theoretical framework for comprehending health-related decision-making through the analysis of perceived susceptibility, severity, benefits, barriers, cues to action, and self-efficacy [10]. The HBM has been extensively used to analyze the behaviors of chronic diseases, but it has not been generally applied to glaucoma treatment preferences, especially in settings with limited resources such as Cambodia.

Thus, this study aimed to examine both clinical and psychosocial factors associated with patient preference for surgical intervention compared with medical therapy among Cambodian adults with advanced glaucoma, guided by the HBM. 

## Materials and methods

Study design and setting

This cross-sectional analytical study was conducted in the Department of Ophthalmology, Preah Ang Duong Hospital, Phnom Penh, Cambodia. As the national tertiary referral center for ophthalmology, Preah Ang Duong Hospital serves patients from all provinces via direct self-referral, non-governmental eye care programs, and public hospitals. The study was conducted over a six-month duration, spanning from January 1 to June 30, 2024.

Study population and eligibility criteria

Participants included adult patients aged 18 years or older who had a confirmed diagnosis of advanced glaucoma in at least one eye. Advanced glaucoma was characterized in accordance with the European Glaucoma Society criteria as the presence of glaucomatous optic neuropathy accompanied by corresponding visual field defects affecting both hemifields or having loss within 5 degrees of fixation on standard automated perimetry.

Exclusion criteria for patients were the presence of secondary glaucoma (neovascular, uveitic, steroid-induced, or traumatic), previous bilateral glaucoma surgery, complete blindness due to glaucoma, significant ocular comorbidities impacting visual function (e.g., advanced diabetic retinopathy or macular degeneration), cognitive impairment that impaired reliable interview responses, and refusal to provide informed consent.

Sample size determination

The required sample size was calculated using Cochran's formula for cross-sectional studies. Based on an estimated proportion of 45.9% preference for surgical intervention, which represents the primary outcome of interest in this study, a 95% confidence level, and a margin of error of 5%, the minimum required sample size is determined to be 381 participants. A target sample size of at least 419 participants was established after accounting for an expected non-response rate of 10%. Eligible patients attending the glaucoma clinic were consecutively recruited during the study period until the necessary sample size was reached.

Although the primary objective of this study was to identify the association with treatment preference, the sample size was calculated based on the estimated prevalence of preference for surgical intervention, as this was the key outcome variable in this cross-sectional analytical study. This approach is consistent with sample size estimation for studies examining associations with a binary outcome in similar settings. 

Sampling technique

A consecutive sampling approach was employed, and all eligible patients attending the glaucoma outpatient clinic throughout the study period were invited to participate. Recruitment occurred daily until the target sample size was achieved.

Data collection procedures

Data were gathered via structured face-to-face interviews and the examination of medical records. Interviews were not audio-recorded; responses were documented in real time using structured questionnaires by trained interviewers in a private room at the outpatient department to guarantee participant comfort and confidentiality. The research team used a standardized data collection form to gather clinical data from patient records.

Study instruments and measurements

Sociodemographic and Clinical Characteristics

Data regarding age, sex, residence, educational level, occupation, health insurance status, duration of glaucoma diagnosis, type of glaucoma, number of prescribed antiglaucoma medications, and history of previous ocular surgery were gathered.

Assessment of Glaucoma Knowledge

A 10-item questionnaire based on previously validated tools was used to measure knowledge of glaucoma. Items measured knowledge of the chronicity of the condition, blindness risk, intraocular pressure control, treatment objectives, and the need for long-term monitoring. Each correct response received one point, and total scores were converted to percentage scores. Higher scores reflect an improved understanding of glaucoma.

HBM Constructs

Health beliefs were evaluated through items derived from the HBM, encompassing perceived susceptibility, perceived severity, perceived benefits of surgery, perceived barriers to surgery, self-efficacy, and cues to action. A five-point Likert scale, spanning from strongly disagree to strongly agree, was used for measuring responses. A composite score was given to each construct, with higher scores meaning more support for the belief in question.

Outcome Variable

The main outcome variable was patient preference regarding glaucoma treatment, classified as preference for surgical intervention or preference for ongoing medical therapy. Preference was measured by asking individuals explicitly which treatment they would select if their ophthalmologist advised it, with the assumption that surgery would primarily try to control disease progression rather than restore lost eyesight.

Data Quality Assurance

To ensure content and face validity, the questionnaire was developed based on previously validated instruments and reviewed by three glaucoma specialists and two public health researchers. The questionnaire was translated into Khmer and back-translated into English by independent bilingual translators to ensure linguistic and conceptual equivalence (Appendices). 

A pilot study was conducted to assess clarity, feasibility, and internal consistency. Based on pilot testing, minor wording adjustments were made to improve comprehension. The internal consistency of the HBM constructs demonstrated acceptable reliability (Cronbach's alpha >0.70 for all domains). 

Data collectors underwent standardized training prior to the start of the study. Daily reviews of completed questionnaires were conducted to ensure completeness and consistency. Double data entry was carried out to reduce transcription errors. Incomplete or missing responses were handled using listwise deletion or multiple imputation, ensuring that analyses included only complete data for each construct. 

Statistical analysis

Data were input into a secure database and analyzed using Stata Version 14.2 (StataCorp LLC, College Station, Texas, United States). Descriptive statistics summarized participant characteristics and study variables. Continuous variables were evaluated for normality and summarized using means with standard deviations or medians with interquartile ranges, as applicable. Categorical variables were presented as frequencies and percentages. Bivariate analyses used chi-squared tests for categorical variables and independent-samples t-tests for continuous variables to compare participants who preferred surgery with those who preferred medical treatment. Variables that had a p-value less than 0.20 in the bivariate analysis were included in more complex models to identify factors that independently influenced the choice of surgical intervention. Adjusted odds ratios (aORs) accompanied by 95% confidence intervals (CIs) were presented. Statistical significance was set at a two-sided p-value of <0.05.

Ethical approval and informed consent

The study protocol received review and approval from the National Ethics Committee for Health Research of the Ministry of Health (MoH) (approval number: 331/23; date: November 2, 2023). The research complied with the tenets of the Declaration of Helsinki. All participants submitted written informed consent prior to enrollment. Participation was voluntary, and patients were informed that declining to participate would not impact their clinical care. Data collected were anonymized before analysis to ensure confidentiality. All data were discarded at the end of the study.

## Results

Participant enrollment, non-response rate, and baseline characteristics

A total of 512 patients diagnosed with advanced glaucoma were recruited during the study period. Twenty-five patients opted out of participation or provided incomplete responses, resulting in a final analytic sample of 487 participants, which corresponds to a response rate of 95.1%. Participants ranged in age from 18 to 86 years, with a mean age of 52.87±14.94 years. Females comprised 239 (49.08%) of the study population. Two hundred and seventy-five (56.47%) participants resided in rural or semi-rural areas, and 338 out of 487 participants (69.40%) had enrolled in national health insurance schemes. In terms of educational attainment, merely eight of them (1.64%) lacked formal education, whereas 51 (10.47%) had not yet completed primary education (see Table 1).

**Table 1:** Demographic characteristics Source: Authors' own data from the cross-sectional survey conducted at Preah Ang Duong Hospital between January and June 2024

Demographic data	Description	
	n	Percentage
Gender		
Male	248	50.92%
Female	239	49.08%
Age (mean±SD)		
≤40	106	21.77%
41-50	76	15.61%
51-60	131	26.90%
61-70	125	25.67%
70+	49	10.06%
Education level		
University graduate	72	14.78%
High school	277	56.88%
Secondary school	79	16.22%
Primary school	51	10.47%
Illiterate	8	1.64%
Job status		
Employed	316	64.89%
Unemployed	60	12.32%
Retired	99	20.33%
Others	12	2.46%
Residence		
Urban	212	43.53%
Rural	275	56.47%
Type of payment		
Indigent	23	4.72%
Paying	126	25.87%
Health Equity Fund	109	22.38%
National Social Security Fund	229	47.02%

Clinical characteristics and treatment

The average number of years of treatment is 2.18±1.90, while the median time after glaucoma diagnosis was 1.5 years. During the interview, 357 of the participants (73.31%) were using two or more topical antiglaucoma medications, while 129 (26.49%) had a history of unilateral glaucoma surgery or any glaucoma-related surgical interventions, including combined phacoemulsification, goniosynechialysis, etc. More details are outlined in Table 2.

**Table 2:** Baseline clinical characteristics of the participants Source: Authors' own data from the present study (n=487)

Clinical data	Description	
	n	Percentage
Type of glaucoma		
Open-angle glaucoma	185	37.99%
Angle-closure glaucoma	153	31.42%
Uveitic or inflammatory glaucoma	67	13.76%
Others (neovascular glaucoma, etc.)	50	10.27%
Angle recession or post-trauma glaucoma	14	2.87%
Pseudo-exfoliative glaucoma	10	2.05%
Lens-related glaucoma	8	1.64%
Years of treatment		
<5	455	93.43%
≥5	32	6.57%
No. of drugs used		
1	130	26.69%
2	110	22.59%
3	106	21.77%
More than 3	141	28.95%
Type of surgical procedures		
Glaucoma tube implant	3	0.62%
Others (phaco-goniosynechialysis, etc.)	35	7.19%
Trabeculectomy +/- mitomycin-C	91	18.69%
None	358	73.51%
Other comorbidities		
Hypertension	259	53.18%
Cataract	154	31.62%
Diabetes	102	20.94%
Dyslipidemia	38	7.80%
Ischemic heart disease	36	7.39%
Obesity	31	6.37%
Hypothyroidism	10	2.05%
Asthma	3	0.62%
Renal disease	2	0.41%
Others	2	0.41%

Primary open-angle glaucoma constituted the predominant diagnosis among 185 patients (37.99%), with primary angle-closure glaucoma following at 153 (31.42%), while other types comprised 149 (21.39%). During the interview, 358 of the patients (73.51%) had not received any surgical treatment for their glaucoma; 91 patients (18.69%) had undergone trabeculectomy with mitomycin-C; 35 patients (7.19%) had other procedures, such as phaco-goniosynechialysis or phaco-trabeculectomy; and only three patients (0.62%) had a glaucoma tube implant.

The patients list hypertension (53.18%; n=259), cataract (31.62%; n=154), and diabetes (20.94%; n=102) as their top three comorbidities. They also list dyslipidemia (7.80%; n=38), ischemic heart disease (7.39%; n=36), obesity (6.37%; n=31), hypothyroidism (2.05%; n=10), asthma (0.62%; n=3), renal disease (0.41%; n=2), and other conditions (0.41%; n=2). The type of glaucoma significantly influenced patient preference. This relationship was statistically significant (χ2=17.088; p=0.009). A significant proportion of patients (79.3%) indicated consistent attendance at clinic follow-ups; however, 41.2% admitted to having missed at least one scheduled appointment in the past year.

Preference for glaucoma treatment modality

**Figure 1:**
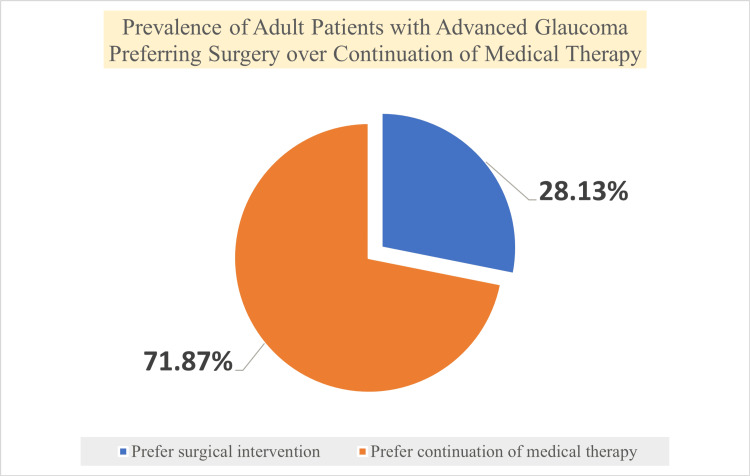
Prevalence of adult patients preferring surgery over continued medical therapy Source: Authors' own data from the cross-sectional study

Overall, 350 individuals (71.87%) favored ongoing medicinal therapy, while 137 participants (28.13%; 95% CI: 24.1-32.2%) indicated a preference for surgical intervention if advised by their ophthalmologist (Figure 1). Preference for surgery was greater among patients with longer disease duration, increased medication burden, and previous glaucoma surgery exposure, although these associations did not maintain significance after multivariable adjustment.

Glaucoma knowledge scores

The cohort as a whole had a mean glaucoma knowledge score of 55.2%±12.6%. Participants who favored surgical intervention exhibited notably higher knowledge scores than those who preferred medical therapy (58.03%±16.13% vs. 54.09%±17.17%; p=0.048); see Table 3. Knowledge gaps were particularly evident concerning the irreversibility of vision loss and the long-term objectives of surgical intervention.

**Table 3:** Relation between score of patient knowledge and his/her preference towards surgical treatment versus medical therapy (n=487) U: Mann-Whitney U test Source: Data collected and analyzed by the authors as part of this study

Do you prefer surgical treatment over continuation of medical therapy?				
	Yes (n=137; 28.13%)	No (n=350; 71.87%)		
Patient's knowledge			U	P-value
Total score: mean±SD	5.80±1.61	5.41±1.72	21221	0.048
%score: mean±SD	58.03±16.13	54.09±17.17		

HBM constructs

Participants who favored surgical treatment had significantly higher mean scores for perceived severity and susceptibility to glaucoma-related blindness. Additionally, they showed increased self-efficacy in handling postoperative care and higher perceived advantages of surgery. Conversely, perceived barriers, specifically fear of surgery, cost concerns, and fear of postoperative vision loss, were significantly greater among participants who preferred medical therapy (all p<0.01; Table 4). Participants who preferred surgical intervention more frequently reported cues to action, including recommendations from ophthalmologists and testimonials from other patients who had undergone surgery (Table 5).

**Table 4:** Relation between health belief domains and patient preference towards surgery versus medical therapy (n=487) U: Mann-Whitney U test Source: Data collected and analyzed by the authors as part of this study

Do you prefer surgical treatment over continuation of medical therapy?				
	Yes (n=137; 28.13%)	No (n=350; 71.87%)		
Health Belief Model			U	P-value
(1) Perceived seriousness				
Total score: mean±SD	8.42±1.73	3.20±0.85	2457	<0.00001
%score: mean±SD	93.59±19.23	35.56±9.44		
(2) Perceived susceptibility				
Total score: mean±SD	8.45±1.54	3.43±1.10	2118	<0.00001
%score: mean±SD	93.84±17.15	38.13±12.27		
(3) Perceived benefits				
Total score: mean±SD	11.07±1.69	6.16±2.28	3216	<0.00001
%score: mean±SD	92.21±14.05	51.33±19.01		
(4) Perceived barriers				
Total score: mean±SD	9.61±3.23	13.23±2.27	9241	<0.00001
%score: mean±SD	19.01±21.53	88.23±15.13		
(5) Self-efficacy				
Total score: mean±SD	7.99±1.79	4.07±1.53	4149	<0.00001
%score: mean±SD	88.73±19.94	45.27±17.01		

**Table 5:** Relation between cutes to action and patient preference for surgical management versus medical therapy (n=137 vs. 350) Source: Data collected and analyzed by the authors as part of this study

		Preferring surgery	Not preferring surgery	Total	Chi-square	P-value
Do you have a family member or a friend who lost vision from glaucoma in spite of the use of medication?	Yes	89	180	269	7.2949	0.007
	No	48	170	218		
Do you have family members or friends who went for successful glaucoma surgery?	Yes	89	196	285	3.2589	0.071
	No	48	154	202		
Does your doctor prompt surgery than medication?	Yes	107	270	377	0.0518	0.821
	No	30	80	110		
Did you get any information from media or social contacts that encourage surgery?	Yes	77	169	246	2.4698	0.116
	No	60	181	241		

Bivariate analysis and multivariable logistic regression analysis

In bivariate analysis, significant associations were found between preference for surgical intervention and higher glaucoma knowledge scores, greater perceived severity, greater perceived benefits, higher self-efficacy, and lower perceived barriers (p<0.05 for all). Sociodemographic variables, including age, sex, residence, education level, and insurance status, did not show a significant association with treatment preference (Table 6).

**Table 6:** Relation between demographic and clinical data to treatment preference (n=487) Source: Data collected and analyzed by the authors as part of this study

Demographic and clinical characteristics	Preferring surgery (n=137; 28.13%)		Not preferring surgery (n=350; 71.87%)		Chi-square	P-value
	n	Percentage	n	Percentage		
Gender						
Male	63	45.99%	176	50.29%	0.7286	0.393
Female	74	54.01%	174	49.71%		
Education level						
University graduate	20	14.60%	52	14.86%	6.5875	0.159
High school	89	64.96%	188	53.71%		
Secondary school	15	10.95%	64	18.29%		
Primary school	11	8.03%	40	11.43%		
Illiterate	2	1.46%	6	1.71%		
Job status						
Employed	87	63.50%	229	65.43%	1.1709	0.761
Unemployed	18	13.14%	42	12%		
Retired	30	21.90%	69	19.71%		
Others	2	1.46%	10	2.86%		
Residence						
Urban	52	37.96%	160	45.71%	2.4108	0.121
Rural	85	62.04%	190	54.29%		
Type of payment						
Indigent	6	4.38%	17	4.86%	2.4456	0.485
Paying	35	25.55%	91	26%		
Health Equity Fund	25	18.25%	84	24%		
National Social Security Fund	71	51.82%	158	45.14%		
Type of glaucoma						
Open-angle glaucoma	49	35.77%	136	38.86%	17.0882	0.009
Angle-closure glaucoma	59	43.07%	94	26.86%		
Uveitic or inflammatory glaucoma	17	12.41%	50	14.29%		
Others (neovascular glaucoma, etc.)	7	5.11%	43	12.29%		
Angle recession or post-trauma glaucoma	1	0.73%	13	3.71%		
Pseudo-exfoliative glaucoma	2	1.46%	8	2.29%		
Lens-related glaucoma	2	1.46%	6	1.71%		
Type of surgical procedures						
Glaucoma tube implant	3	2.19%	0	0%	0.5072	0.776
Others (phaco-goniosynechialysis, etc.)	32	23.36%	3	0.86%		
Trabeculectomy +/- mitomycin-C	81	59.12%	10	2.86%		
None	21	15.33%	237	67.71%		

Variables that satisfied the inclusion criteria from the bivariate analysis were incorporated into a multivariable logistic regression model. Following adjustment, four constructs of the HBM were found to be independently associated with the preference for surgical intervention: perceived severity (aOR: 1.71; 95% CI: 1.17-2.51), perceived benefits of surgery (aOR: 1.51; 95% CI: 1.16-1.94), self-efficacy (aOR: 1.46; 95% CI: 1.11-1.93), and perceived barriers (aOR: 0.61; 95% CI: 0.49-0.77). Following adjustment for health belief constructs, the glaucoma knowledge score did not demonstrate an independent association with surgical preference (Table 7).

**Table 7:** Univariate and multivariate logistic regression analysis affecting patient preference for surgical management versus medical therapy (n=137 vs. 350) *Reference value Source: Data collected and analyzed by the authors as part of this study

	Univariate		Multivariate	
	OR (95% CI)	P-value	OR (95% CI)	P-value
Health Belief Model domains				
Perceived seriousness	1.64 (1.15 to 2.33)	0.006	1.71 (1.17 to 2.51)	0.008
Perceived susceptibility	1.38 (0.95 to 2.01)	0.082	1.34 (0.91 to 1.99)	0.129
Perceived benefits	1.50 (1.17 to 1.92)	0.001	1.51 (1.16 to 1.94)	0.001
Perceived barriers	0.64 (0.52 to0.79)	<0.001	0.61 (0.49 to 0.77)	<0.0001
Self-efficacy	1.45 (1.10 to 1.89)	0.007	1.46 (1.11 to 1.93)	0.006
Gender				
Male	1*		1*	
Female	1.19 (0.79 to 1.76)	0.394	1.81 (0.54 to 6.16)	0.337
No. of drugs	1.05 (0.90 to 1.23)	0.515	0.78 (0.48 to 1.26)	0.313
Level of knowledge	1.15 (1.02 to 1.29)	0.022	1.36 (0.91 to 2.01)	0.135
Age	1.02 (1.00 to 1.02)	0.038	1.01 (0.97 to 1.05)	0.484

## Discussion

Using the HBM as an explanatory framework, this study offers one of the earliest comprehensive and extensive analyses of glaucoma treatment preference among people with advanced disease, if not in Cambodia, in our resource-constrained clinical setting. The findings indicate that the preference for surgical intervention is low, with less than one-third of patients expressing a willingness to undergo surgery, even in the context of advanced disease severity. Treatment preference was primarily influenced by psychosocial factors rather than demographic or clinical variables, highlighting the significant impact of patient beliefs on glaucoma decision-making.

Interpretation of key findings through the HBM

The strong association between perceived severity and preference for surgical intervention supports the HBM hypothesis that individuals are more likely to adopt health-protective behaviors when they recognize the seriousness of their condition [11]. Patients who understood the irreversible nature of glaucomatous visual loss and the limitations of conventional therapy were more likely to accept surgery. This finding aligns with research from high-income and middle-income contexts, indicating that perceived disease severity significantly influences the acceptance of invasive interventions in chronic eye diseases [8,12].

The perceived benefits of surgery were identified as a significant predictor of surgical preference. Patients who believed that surgery could achieve sustained control of intraocular pressure and decrease long-term reliance on medication demonstrated a significantly higher preference for surgical management [8]. This is consistent with previous studies showing that patients frequently underestimate the long-term advantages of glaucoma surgery, especially when visual acuity is largely maintained [8,13,14]. Ignoring these advantages could delay the acceptance of final therapy in advanced glaucoma, where functional vision loss may occur before vision loss.

Conversely, perceived barriers, such as fear of surgical intervention, financial considerations, and anxiety about postoperative vision impairment, were strongly linked to a preference for ongoing medical treatment. Barriers have been consistently acknowledged in low- and middle-income countries, where restricted access to surgical services, indirect costs, and sociocultural beliefs regarding eye surgery affect how patients choose [15,16]. In Cambodia, significant out-of-pocket health expenditures persist despite the existence of national insurance schemes, suggesting that financial barriers significantly influence treatment preferences.

Additionally, self-efficacy also independently predicted preference for surgical intervention. Patients who felt confident in their ability to undergo surgery, adhere to postoperative care, and attend follow-up visits were more willing to consider surgical treatment. This finding underscores the significance of patient empowerment and education in managing chronic diseases, aligning with evidence from studies on glaucoma and cataract care that indicate a correlation between higher self-efficacy and enhanced treatment uptake and adherence [8,17].

Glaucoma knowledge and its indirect role

Higher glaucoma knowledge scores were initially linked to a preference for surgery; however, this association did not remain significant after adjusting for HBM constructs. This shows that knowledge alone is insufficient to influence treatment decisions in our populations unless it results in significant changes in perceived severity, benefits, and barriers. Information provided without addressing emotional and contextual aspects has been found to have little effect on behavior in other chronic eye disorders [8,17-19]. The findings underscore the necessity for counseling strategies that extend beyond factual education and involve beliefs, fears, and expectations.

Comparison with previous literature

The low surgical preference documented in this study contrasts with findings from high-income countries, where earlier diagnosis, greater confidence in healthcare systems, and more comprehensive insurance coverage led to increased acceptability of glaucoma surgery [19]. The results, however, are in line with research from sub-Saharan Africa and Southeast Asia, where even in cases of severe disease, late presentation and reliance on medical care are typical [15,20]. The HBM's cross-cultural applicability in glaucoma care was further supported by a study conducted among glaucoma patients in Egypt, which found that perceived advantages and barriers were the best indicators of surgical acceptability [8]. This study contributes to the existing literature by offering context-specific evidence from Cambodia, a region where empirical data on glaucoma decision-making is limited.

Clinical and public health implications

These findings are pertinent to the management of glaucoma within our routine care practices and may also be applicable in Cambodia by highlighting the significance of integrating structured, patient-centered counseling into standard glaucoma treatment protocols. Counseling should explicitly address misconceptions related to surgery, emphasize the irreversible nature of glaucomatous damage, and set realistic expectations regarding surgical results. Interventions designed to address perceived barriers, such as financial counseling, peer-support initiatives, and testimonials from patients who have successfully undergone treatment, may improve acceptance of surgical procedures. Third, bolstering self-efficacy through optimized postoperative care pathways and enhanced follow-up support may facilitate prompt surgical intervention. These results endorse the incorporation of behavioral health frameworks into national eye care policies at the policy level. Addressing the psychosocial determinants influencing care-seeking behavior is essential for attaining universal eye health and reducing preventable blindness caused by glaucoma [21].

Limitations

This investigation is characterized by several limitations. The cross-sectional design restricts the ability to establish causality, and expressed treatment preferences may not necessarily reflect actual behavior. Data were collected at a single tertiary center, potentially limiting the generalizability to primary or secondary care settings, as well as during ongoing community-based care. Health belief constructs were evaluated using self-administered questionnaires, which may be subject to social desirability bias. Despite these limitations, the large sample size and the theory-based analytical approach strengthen the validity and relevance of the findings.

## Conclusions

Within our clinical practice, the inclination toward surgical treatment among Cambodian patients with advanced glaucoma remains markedly minimal and is predominantly influenced by health-related beliefs rather than exclusively by demographic or clinical considerations. Enhancing glaucoma management outcomes necessitates the implementation of strategies that encompass psychosocial support, patient education, and the mitigation of barriers within the healthcare system.

## Appendices

**Table 8:** Questionnaire MIGS: minimally invasive glaucoma surgery; IHD: ischemic heart disease; IOP: intraocular pressure; OCT: optical coherence tomography; QoL: quality of life; HBM: Health Belief Model

Section I. Sociodemographic and clinical characteristics				
Item code	Domain	Question/variable	Response options (code)	Scale type
a00	Sociodemographic	Type of participant	Paying (1); Indigent (2); Insurance coverage (3); Health Equity Fund (4); National Social Security Fund (5)	Nominal
a01	Sociodemographic	Type of payment	Full (1); Partial (2)	Nominal
a02	Sociodemographic	Age (years)	Continuous	Continuous
a03	Sociodemographic	Sex	Female (1); Male (2)	Nominal
a04	Sociodemographic	Education level	University graduate (1); High school (2); Primary/secondary (3); Illiterate (4)	Ordinal
a05	Sociodemographic	Residence	Urban (1); Rural (2)	Nominal
a06	Sociodemographic	Occupation status	Employed (1); Unemployed (2); Retired (3); Other (4)	Nominal
a07	Clinical	Type of glaucoma	Open-angle (1); Angle-closure (2); Pseudo-exfoliative (3); Post-traumatic (4); Uveitic (5); Lens-related (6); Other (7)	Nominal
a08	Clinical	Duration of treatment (months)	Continuous	Continuous
a09	Clinical	Number of drugs used	1-5	Discrete
a10	Clinical	Surgery recommended	Trabeculectomy (1); Tube implant (2); MIGS (3); Other (4)	Nominal
a11	Clinical	Underwent glaucoma surgery	Yes (1); No (2)	Binary
a12	Clinical	Type of surgery performed	Trabeculectomy (1); Tube implant (2); MIGS (3); Other (4)	Nominal
a13	Clinical	Comorbidities (multiple answers)	Cataract; Hypertension; Diabetes; Obesity; Dyslipidemia; IHD; Renal disease; Hypothyroidism; Asthma; Other	Nominal
Section II. Knowledge about glaucoma				
Item code	Statement	Response options (code)	Scoring	
b01	Glaucoma is curable	True (1); False (2)	1=Correct	
b02	Siblings of glaucoma patients are at risk	True (1); False (2)	1=Correct	
b03	Normal intraocular pressure (mmHg)	5-10 (1); 10-20 (2); ≥20 (3); Don’t know (4)	1=Correct	
b04	All glaucoma patients have high IOP	True (1); False (2)	1=Correct	
b05	Investigation required for follow-up	Ultrasound (1); OCT (2); Visual field (3); Don't know (4)	1=Correct	
b06	Effective treatment will	Slow progression (1); Regenerate nerve (2); Don't know (3)	1=Correct	
b07	Alternate management options	Surgery (1); Laser (2); Both (3); None (4); Don't know (5)	1=Correct	
b08	IOP may decrease after cataract surgery	True (1); False (2)	1=Correct	
b09	Side effects of glaucoma drugs	Multiple responses	1=Correct	
b10	Steroids may decrease IOP	True (1); False (2)	1=Correct	
Total knowledge score: sum of correct responses				
Section III. Patient preferences				
Item code	Question	Response options (code)		
c01	Preference for surgery over continued medical therapy	Yes (1); No (2)		
Section IV. Health Belief Model Domains				
Item code	Domain	Question/variable	Response options (code)	Scale type
Item code	Statement			
d01	HBM – Seriousness	Medications cause irritating side effects	Disagree (1); Neutral (2); Agree (3)	Likert (1-3)
d02	HBM – Seriousness	Medications interfere with daily activity	Disagree (1); Neutral (2); Agree (3)	Likert
d03	HBM – Seriousness	Lifelong medication affects quality of life	Disagree (1); Neutral (2); Agree (3)	Likert
d04	HBM – Susceptibility	Difficult to adhere to lifelong medication	Disagree (1); Neutral (2); Agree (3)	Likert
d05	HBM – Susceptibility	Vision may worsen despite adherence	Disagree (1); Neutral (2); Agree (3)	Likert
d06	HBM – Susceptibility	Medication requires more follow-up	Disagree (1); Neutral (2); Agree (3)	Likert
d07	HBM – Benefits	Surgery is a permanent solution	Disagree (1); Neutral (2); Agree (3)	Likert
d08	HBM – Benefits	Freedom from medication improves QoL	Disagree (1); Neutral (2); Agree (3)	Likert
d09	HBM – Benefits	Surgery minimizes vision damage	Disagree (1); Neutral (2); Agree (3)	Likert
d10	HBM – Benefits	Surgery more effective than medication	Disagree (1); Neutral (2); Agree (3)	Likert
d11	HBM – Barriers	Surgery may damage the eye	Disagree (1); Neutral (2); Agree (3)	Likert
d12	HBM – Barriers	Surgery may worsen condition	Disagree (1); Neutral (2); Agree (3)	Likert
d13	HBM – Barriers	Surgery is unaffordable	Disagree (1); Neutral (2); Agree (3)	Likert
d14	HBM – Barriers	Medical therapy is safer than surgery	Disagree (1); Neutral (2); Agree (3)	Likert
d15	HBM – Barriers	General health unfit for surgery	Disagree (1); Neutral (2); Agree (3)	Likert
d16	HBM – Cues	Family/friend lost vision despite medication	Yes (1); No (2)	Binary
d17	HBM – Cues	Family/friend had successful surgery	Yes (1); No (2)	Binary
d18	HBM – Cues	Doctor recommends surgery	Yes (1); No (2)	Binary
d19	HBM – Cues	Media/social contacts encourage surgery	Yes (1); No (2)	Binary
d20	HBM – Self-efficacy	Able to discuss surgery with physician	Disagree (1); Neutral (2); Agree (3)	Likert
d21	HBM – Self-efficacy	Able to withstand surgical stress	Disagree (1); Neutral (2); Agree (3)	Likert
d22	HBM – Self-efficacy	Able to comply with perioperative care	Disagree (1); Neutral (2); Agree (3)	Likert
